# From Felix, Cross Roads

**Published:** 1882-10

**Authors:** 

**Affiliations:** Cross Roads


CROSS ROADS, September 12, 1882.

*Editor* of DENTAL REGISTER: Did you ever notice that as funny things occur in reporting, as are found in the "tricks of the types?"

Look at page 437, in the REGISTER for September: "We do know how some medicines act, and we don't know how to harness others." Don't know "*as to others*" was the expression used; but Watt is hard to report.

As I have begun, may I notice a few points in your report? On page 439, Prof. Mayr presents as an objection to Dr. Watt's theory of arsenious acid destroying the vitality of lower organisms, that mold was found in his laboratory, though a vial containing a solution of arsenious acid, and another containing a solution of arsenite of soda stood uncorked therein. He regards this as positively contradicting Dr. Watt's position. But what if the vials were uncorked? The water in them might evaporate, but the poisons would remain in the vials. If his laboratory had been permeated, throughout, by absorbent vessels, and small tubes to promote capillary attraction, the poisons would have had some chance to kill the mold. But unless each vial possessed the skill of a sportsman, and had a shot-gun, they were helpless under the circumstances. I was taught that chemical action requires contact. Affinity is powerless at long range.

And do you know why Profs. Rawls and Mayr regard the term "vital force" as an empty word? I would not like to do without either the name or the essence.

On page 433, "Voice" asks "Is it not possible that the sanguinary calculus may derive some, or part of the constituents from the breaking down of the alveolus?"

Certainly it is possible; and when a dewdrop evaporates from a roseleaf near Windsor Castle, it is *possible* that it will settle down into the reservoir of the water-works and quench the thirst of Queen Victoria. But the materials of the dissolved alveolus go into the general circulation, and are used accordingly. The idea that they would be deposited at the nearest points, is a labor-saving thought not known in physiology.

On page 443, it appears, from your report, that Dr. Hurtt was *hurt* at seeing the American Dental Association assume, to some extent, the character of a Peddlers' Exchange. I happened to be out at the time, or I might have been hurt too. Don't like to see an association forced to turn a power grindstone for sharpening private axes. Is that the idea? But I fear I have over-taxed your space and patience. Respectfully, FELIX.

